# Supplementary material for: NMR Structure of the FIV gp36 C-terminal Heptad Repeat and Membrane-Proximal External Region
Source: Int J Mol Sci. 2020 Mar 16;21(6):2037. doi: 10.3390/ijms21062037 (PMC7139756; doi:10.3390/ijms21062037)

Table of Contents:

Fig. S1: A) ^{15}N-HSQC NMR spectrum of (^{15}N-^{13}C)-labeled gp36-MPER in DPC/SDS 90:10 M/M mixed micelles; B) folded ^{13}C-HSQC NMR spectrum of (^{15}N-^{13}C)-labeled gp36-MPER in DPC/SDS 90:10 M/M mixed micelles.

Table S1: TALOS+ prediction of gp36 CHR-MPER backbone dihedral angles (PHI, PSI), estimated standard deviations of the prediction errors in PHI and PSI (DPHI, DPSI), TALOS+ database matching score (DIST), Wishart RCI chemical shift order parameter (S2), number of database triplets used to form the torsion angle predictions (COUNT, CS COUNT), classification of the prediction result (CLASS; **None** if no torsion prediction was made, **Good** if majority consensus in database matches, **Warn** if no consensus in database matches, **Dyn** if RCI S2 value indicates dynamic conformation).

Table S2: ^{1}H, ^{15}N and ^{13}C chemical shift of (^{15}N-^{13}C)-labeled gp36-MPER in DPC/SDS 90:10 M/M mixed micelles**.**

Fig. S2: A) FPLC chromatogram ; B) HPLC chromatogram, obtained using gradient: 10-40 B in 20 min poi 40-70 B in 30 min, Buffer A: water + TFA 0.1%, Buffer B: Acetonitril + TFA 0.1%; C) SDS-PAGE of FPLC fractions (4-5), after cleavage (6) and HPLC fractions (7 KSI - 8 isotope labeled gp36-MPER).

Figure S1: A) ^{15}N-HSQC NMR spectrum of (^{15}N-^{13}C)-labeled gp36-MPER in DPC/SDS 90:10 M/M mixed micelles; B) folded ^{13}C-HSQC NMR spectrum of (^{15}N-^{13}C)-labeled gp36-MPER in DPC/SDS 90:10 M/M mixed micelles.

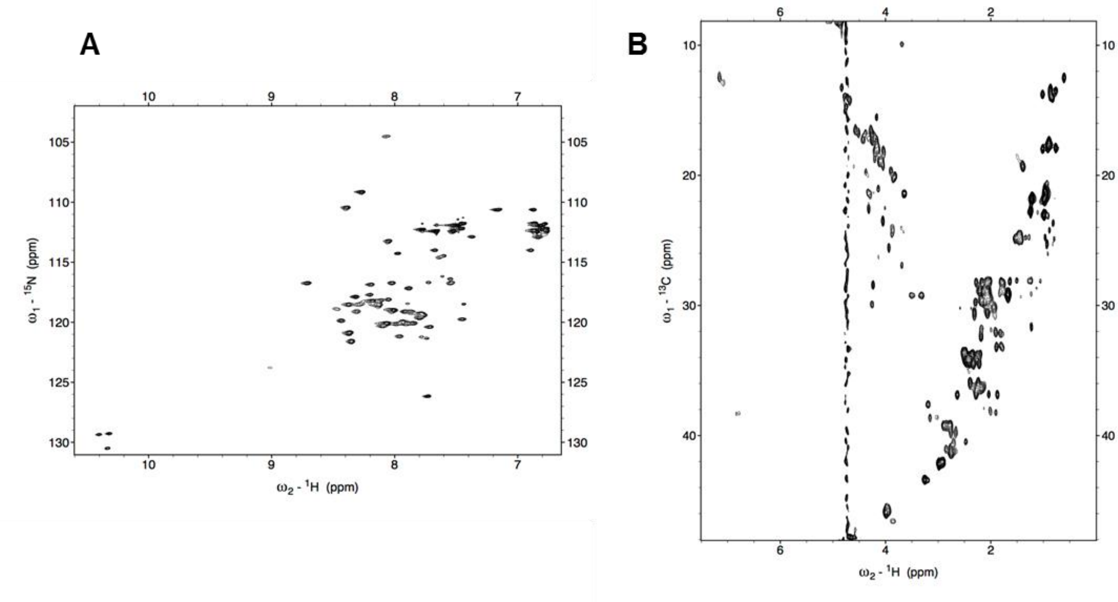

Table S1: TALOS+ prediction backbone dihedral angles of 0.5 mg (^{15}N-^{13}C)-labeled gp36 CHR-MPER acquired on Bruker 900 MHz in DPC/SDS 90:10 M/M mixed micelles**,** at pH 7.4 and at 300 K. Gp36 CHR-MPER backbone dihedral angles (PHI, PSI), estimated standard deviations of the prediction errors in PHI and PSI (DPHI, DPSI), TALOS+ database matching score (DIST), Wishart RCI chemical shift order parameter (S^2), number of database triplets used to form the torsion angle predictions (COUNT, CS COUNT), classification of the prediction result (CLASS; **None** if no torsion prediction was made, **Good** if majority consensus in database matches, **Warn** if no consensus in database matches, **Dyn** if RCI S^2 value indicates dynamic conformation).

RESID	RESNAME	PHI (°)	PSI (°)	DPHI (°)	DPSI (°)	DIST	S^2	COUNT	CS_COUNT	CLASS
**740**	T	-59.27	-32.743	6.798	6.694	0.295	0.82	25	13	**Strong**
**741**	K	-64.099	-36.692	3.451	5.224	0.198	0.838	25	15	**Strong**
**742**	D	-69.767	-38.96	3.261	4.211	0.139	0.864	25	14	**Strong**
**743**	L	-64.344	-42.725	3.785	3.874	0.119	0.872	25	14	**Strong**
**744**	Q	-61.889	-42.434	2.942	3.434	0.118	0.88	25	14	**Strong**
**745**	Q	-64.923	-41.272	1.866	2.816	0.123	0.875	25	14	**Strong**
**746**	K	-65.752	-40.177	3.738	4.614	0.136	0.875	25	14	**Strong**
**747**	F	-67.009	-36.821	4.576	5.526	0.181	0.88	25	14	**Strong**
**748**	Y	-64.819	-33.798	3.675	6.293	0.265	0.865	25	14	**Strong**
**749**	E	-66.576	-34.207	6.072	6.355	0.479	0.837	25	13	**Strong**
**750**	I	-71.134	-33.281	8.714	9.908	0.685	0.823	10	12	**Generous**
**751**	I	-70.167	-33.55	12.121	10.156	0.628	0.839	25	12	**Strong**
**752**	L	-64.437	-41.439	3.934	4.164	0.499	0.867	25	13	**Strong**
**753**	D	-66.624	-39.424	4.273	3.586	0.333	0.88	25	13	**Strong**
**754**	I	-64.585	-41.763	3.305	3.019	0.218	0.87	25	14	**Strong**
**755**	E	-64.433	-37.712	3.857	7.155	0.188	0.85	25	14	**Strong**
**756**	Q	-66.81	-29.89	4.93	7.798	0.195	0.79	25	15	**Strong**
**757**	N	-72.646	-23.666	8.89	9.49	0.249	0.675	25	15	**Strong**
**758**	**N**	-83.381	-9.776	13.122	14.316	0.301	0.578	10	15	**Dyn**
**759**	**V**	-85.832	-17.811	11.078	12.641	0.347	0.549	7	15	**Dyn**
**760**	**Q**	-59.901	127.866	5.762	5.666	0.377	0.574	25	13	**Dyn**
**761**	**G**	80.971	4.017	10.068	14.434	0.67	0.596	25	13	**Dyn**
**762**	**K**	-119.08	32.449	13.865	27.416	1.519	0.584	5	13	**Dyn**
**763**	**T**	-81.612	138.095	27.123	13.574	-1.319	0.59	25	13	**Dyn**
**764**	**G**	87.54	4.72	9.69	15.78	1.065	0.652	25	12	**Strong**
**765**	I	-83.032	-23.771	21.661	19.913	0.665	0.732	6	12	**Warn**
**766**	Q	-64.897	-37.011	6.741	7.086	0.508	0.797	25	14	**Strong**
**767**	Q	-65.743	-40.861	4.731	5.737	0.497	0.798	25	14	**Strong**
**768**	L	-64.078	-40.361	4.498	4.57	0.558	0.792	25	13	**Strong**
**769**	Q	-67.642	-35.127	4.154	4.969	0.479	0.761	25	13	**Strong**

**770**	K	-67.202	-36.082	5.542	5.681	0.429	0.777	25	13	**Strong**
**771**	W	-70.467	-33.251	11.365	13.761	0.383	0.809	25	14	**Strong**
**772**	E	-63.52	-41.008	5.068	6.5	0.318	0.869	25	14	**Strong**
**773**	D	-67.762	-37.224	4.255	5.09	0.298	0.884	25	15	**Strong**
**774**	W	-64.26	-43.256	3.666	6.78	0.43	0.868	25	14	**Strong**
**775**	V	-65.844	-35.363	7.173	7.915	0.559	0.853	25	11	**Strong**
**776**	G	-65.321	-35.277	4.082	7.93	0.755	0.826	8	10	**Warn**
**777**	W	-65.683	-36.749	5.126	8.095	0.782	0.808	25	10	**Strong**
**778**	**I**	-79.538	-15.175	13.499	16.625	0.862	0.756	25	11	**Strong**
**779**	**G**	-72.287	-19.263	11.195	6.852	0.938	0.708	4	12	**Warn**
**780**	**N**	-95.479	-8.29	13.461	12.633	0.686	0.64	10	12	**Generous**
**781**	**I**	-70.999	136.664	6.686	11.093	0.799	0.627	25	9	**Strong**
**782**	**P**	-58.878	143.966	6.645	8.142	0.811	0.662	25	8	**Strong**
**783**	Q	-59.959	-34.931	3.728	5.313	0.996	0.72	25	8	**Strong**
**784**	Y	-74.76	-18.594	15.94	20.997	0.948	0.727	25	12	**Strong**
**785**	L	-65.858	-30.25	8.291	7.025	0.817	0.715	25	8	**Strong**

Table S2: ^{1}H, ^{15}N and ^{13}C chemical shift of 0.5 mg (^{15}N-^{13}C)-labeled gp36-MPER acquired on Bruker 900 MHz in DPC/SDS 90:10 M/M mixed micelles**.** The final pH was 7.4. NMR experiments were recorded at 300 K.

Number Resudue	Residue	HN	^{15}N	^{13}Cα	Hα	^{13}Cβ	Hβ	Hγ	Others
**738**	**Q**			54.736	4.752	30.450	2.615/2.311		
**739**	**T**	8.752	117.073	65.468	4.290	68.366	4.155		
**740**	**K**	8.387	120.983	58.262	4.217	32.055	1.851		
**741**	**D**	7.796	119.367	56.514	4.610	40.956	2.821		
**742**	**L**	7.741	120.066	58.351	4.020		1.677		QQδ 0.924
**743**	**Q**	8.337	117.844	60.124	3.833	28.693	2.258		
**744**	**Q**	7.932	117.201	58.798	4.134	28.051	2.249		
**745**	**K**	7.929	118.588	58.332	4.177		1.983	1.559	Qδ 1.804
**746**	**F**	8.368	111.615	61.053	4.203	38.576	3.135		Qδ 7.111
**747**	**Y**	8.128	117.472	61.007	4.122	37.938	3.189		Qε 7.115<br>Cε1 92.892
**748**	**E**	8.088	117.921	58.059	4.010		2.081	2.337	
**749**	**I**	7.918	119.996	59.189	4.063		1.896	1.524	Qδ1 0.793
**750**	**I**	7.831	118.343	60.388	3.825		1.919	1.266/1.011	Qδ1 0.830
**751**	**L**	7.938	120.068	62.522	3.999		1.701		QQδ 0.807
**752**	**D**	7.733	119.459	56.789	4.514	41.197	2.711		
**753**	**I**	8.003	120.007	64.227	3.876		1.906	1.080/0.894	
**754**	**E**	8.389	121.040	58.836	4.068	29.435	2.124		
**755**	**Q**	8.221	117.395	57.850	4.133	28.789	2.152		
**756**	**N**	8.127	116.880	54.230	4.663	39.125	2.807		
**757**	**N**	8.238	118.284	53.950	4.748	39.115	2.897/2.730		
**758**	**V**	8.047	119.072	63.518	4.042	32.100	2.195	0.955	
**759**	**Q**	8.405	121.028	56.755	4.255	28.856	2.104/2.017		
**760**	**G**	8.247	108.979	45.809	4.121				
**761**	**K**	8.136	120.069	57.020	4.188	33.020	1.830	1.335	
**762**	**T**	8.101	113.101	61.134	4.338	69.847	4.650		
**763**	**G**	8.437	110.298	45.914	3.963				
**764**	**I**	8.165	120.090	59.331	4.026		1.909	1.420/0.894	
**765**	**Q**	8.420	121.495	58.691	4.098	28.690	2.350/2.062		
**766**	**Q**	8.154	118.402	57.098	4.239	28.641	2.058		
**767**	**L**	7.877	119.893	62.603	4.322		1.738		QQδ 0.807
**768**	**Q**	7.802	119.419	55.532	4.437		1.918/1.813	2.191	
**769**	**K**	7.619	116.117	57.040	4.182	33.064	1.841		
**770**	**W**	8.023	114.881	57.216	4.607		3.411		Hε1 10.512<br>Nε1 123.999
**771**	**E**	8.408	119.529	59.912	3.865	29.285	2.080		
**772**	**D**	8.148	118.450	56.963	4.354	40.357	2.677/2.293		
**773**	**W**	7.932	120.979	61.338	4.352	29.151	3.537/3.300		Hδ1 7.263<br>Hε1 10.424<br>Nε1 123.126
**774**	**V**	8.282	118.522	61.411	3.626		2.131	1.052/0.938	
**775**	**R**	8.097	119.012		4.231				
**776**	**W**	7.865	120.025	61.455	4.288		3.389		Hε1 10.511
**777**	**I**	8.493	118.531	61.471	3.648		2.050	1.484/0.988	
**778**	**G**	8.073	104.570	46.587	3.853				

**779**	**N**	7.412	116.699	53.260	4.826	39.672	2.756/2.678		
**780**	**I**	7.800	121.083	60.065	3.783		2.101	1.663	Qδ1 0.904
**781**	**P**							2.042	Qδ 3.854
**782**	**Q**	7.946	119.632	65.547	3.985		2.021		
**783**	**Y**	7.939	119.882	57.352	4.260		3.154		Qε 7.149<br>Cε1 92.486
**784**	**L**	7.738	116.447	57.971	4.134		1.862		QQδ 0.872
**785**	**K**								
**786**	**M**								

Figure S2: A) FPLC chromatogram obtained using: binding buffer (10 mM Guanidine, 0,5 M NaCl, 20 mM Tris-HCl and 15mM Imidazole) and elution buffer (10 mM Guanidine, 0,5 M NaCl, 20 mM Tris-HCl and 50 mM Imidazole). The protein was purified using His-Trap™ HP column at 1 mL/min and AKTA purifier system. **B)** HPLC chromatogram, obtained using gradient: 10-40 B in 20 min then 40-70 B in 30 min, Buffer A: water + TFA 0.1%, Buffer B: Acetonitril + TFA 0.1%; the protein was purified using C18 Phenomenex™ column at 2 mL/min. **C)** SDS-PAGE of FPLC fractions (4-5), after cleavage (6) and HPLC fractions (7 KSI - 8 isotope labeled gp36 CHR-MPER).

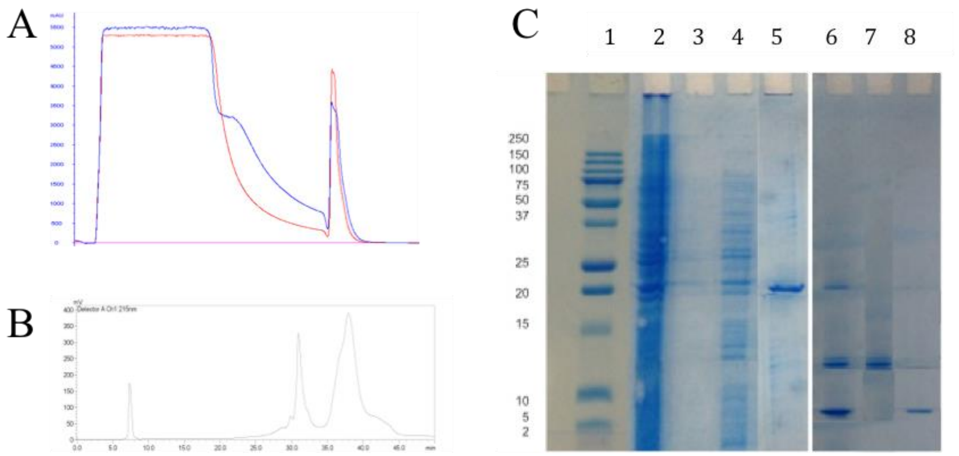

Supplement: Supplementary file 1 [file ijms-21-02037-s001.pdf]
